# Comparative transcriptomic analysis of Tibetan *Gynaephora* to explore the genetic basis of insect adaptation to divergent altitude environments

**DOI:** 10.1038/s41598-017-17051-4

**Published:** 2017-12-05

**Authors:** Qi-Lin Zhang, Li Zhang, Xing-Zhuo Yang, Xiao-Tong Wang, Xiao-Peng Li, Juan Wang, Jun-Yuan Chen, Ming-Long Yuan

**Affiliations:** 1 State Key Laboratory of Grassland Agro-Ecosystems, College of Pastoral Agricultural Science and Technology, Lanzhou University, Lanzhou, 730020 China; 2 Key Laboratory of Grassland Livestock Industry Innovation, Ministry of, Agriculture, China; 3 Evo-devo Institute, School of Life Science, Nanjing University, Nanjing 210023, China; Nanjing Institute of Geology and Paleontology, Nanjing, 210008 China

## Abstract

Adaptation of insects to different altitudes remain largely unknown, especially those endemic to the Tibetan Plateau (TP). Here, we generated the transcriptomes of *Gynaephora menyuanensis* and *G. alpherakii*, inhabiting different high altitudes on the TP, and used these and the previously available transcriptomic and genomic sequences from low-altitude insects to explore potential genetic basis for divergent high-altitude adaptation in *Gynaephora*. An analysis of 5,869 orthologous genes among *Gynaephora* and other three low-altitude insects uncovered that fast-evolving genes and positively selected genes (PSGs) in the two *Gynaephora* species were enriched in energy metabolism and hypoxia response categories (e.g. mitochondrion, oxidation-reduction process, and response to oxidative stress). Particularly, mTOR signaling pathway involving hypoxia was enriched by PSGs, indicating this well-known pathway in mammal hypoxia adaptation may be an important signaling system in *Gynaephora*. Furthermore, some PSGs were associated with response to hypoxia (e.g. cytochrome proteins), cold (e.g. dehydrogenase) and DNA repair (e.g. DNA repair proteins). Interestingly, several insect-specific genes that were associated with exoskeleton and cuticle development (e.g. chitinase and ecdysteroids) had experienced positive selection, suggesting the specific adaptive mechanisms in insects. This study is favourable for understanding the adaptive evolution of *Gynaephora* and even TP insects to divergent altitudes.

## Introduction

The speciation of animals is closely related to different environmental conditions^1,2^, and ecologically divergent selection is an essential evolutionary force in ecological speciation^1,3^. Adaptation to divergent environments could exhibit at the gene sequence variation level; namely, variations in sequences are key targets of natural selection caused by divergent environments^4^. With the wide use of next generation sequencing technologies in ecology and evolution, ecological speciation has been extensively studied in many species systems^2,4^, greatly deepening our understanding of how animals adapt to divergent environments. Currently, RNA sequencing (RNA-seq) has been a useful tool to study ecological adaptation and speciation in natural populations of non-model species^4,5^.

The Tibetan Plateau (TP), with distinctive climate and geography characteristics, is the highest average altitude in the world. Hypoxia, cold climate and high levels of ultraviolet (UV) exposure are gradually enhanced with an altitude gradient, and these major ecological challenges are the most important environmental features on the TP. To adapt to high-altitude environments, animals on the TP have evolved adaptive characteristics in morphology, physiology and behavior^6,7^. Therefore, TP organisms may be satisfactory materials for studying adaptive evolution to divergent ecological environments. In particular, closely related species living in divergent altitude environments provide natural model systems that could be used to explore adaptive mechanisms of organisms to different altitudes. During the past decade, the genetic basis of adaptation to high altitude habitats on the TP has been well documented in many vertebrates, including mammals^8–14^, reptiles^15,16^, birds^17^, amphibians^4^ and fishes^5,18,19^; however, the exploration of high-altitude adaptation in insects has only been conducted for the migratory locust by analyzing gene expression and mitochondrial enzyme activity^20–22^. Currently, how the TP insects adapt to divergent high-altitude environments is still poor understood.

The genus *Gynaephora* (Lepidoptera: Erebidae: Lymantriinae), also known as grassland caterpillars, consists of 15 nominated species in the worldwide. Seven species are mainly distributed in mountainous areas of the Northern Hemisphere and the Arctic tundra, and the other eight species endemic to the TP^23^. The TP *Gynaephora* species are major pests in alpine meadows^23^ and have well adapted to high-altitude environments^23–25^. Our analysis of all the eight TP *Gynaephora* species based on two mitochondrial and two nuclear loci indicated that the diversification and speciation of the TP *Gynaephora* species with a common ancestor occurred within a relatively short period (3.3–0.8 million years ago, Mya) and were associated with the TP uplift and climate changes^24^. Currently, almost all of the TP *Gynaephora* species have elevation specific distributions. *G*. *alpherakii* and *G*. *menyuanensis* are distributed in the highest (~5000 m above sea level (masl)) and lowest altitudes (~3000 masl) among all the eight TP *Gynaephora* species. Our field investigation found that *G*. *alpherakii* showed deeper body color, smaller size, and stronger cold tolerance (lower supercooling point) than *G. menyuanensis* ^26^. Recently, we found that gene sequence variations and expression patterns of mitochondrial genes were associated with adaptive divergence between *G. menyuanensis* and *G. alpherakii* ^25^. Differences of ecological environments caused by different altitudes, with different oxygen content, temperature and intensity of UV radiation, were important driving forces for their divergent altitude adaptation on the TP^25^. However, due to inadequate genetic information on the mitochondrial genome by comparing with nuclear genome, adaptive mechanisms remain largely unknown. Therefore, further exploration for sequence variations of nuclear protein-coding genes is essential for uncovering adaptive evolution of the two *Gynaephora* species to divergent altitude environments.

In this study, we sampled whole developmental stages (including egg, 1–6 larva instar, pupa and female adult) for *G. alpherakii* and *G. menyuanensis*, and then sequenced the transcriptomes for each species. Subsequently, we *de novo* assembled the transcriptomes of *G. alpherakii*, *G. menyuanensis* and *Lymantria dispar* (a species of Lymantriinae living in plain). We performed a comparative transcriptomic analysis for the two *Gynaephora* species together with other three species of insects (*L. dispar, Bombyx mori, Manduca sexta*) living in low-altitude region to explore the adaptive signals in gene sequences that may be associated with ecological adaptation and speciation due to divergent high-altitude environments. We identified a set of fast-evolving genes (FEGs) and positively selective genes (PSGs), and especially focused on PSGs involving response to hypoxia, cold, strong UV radiation and insect-specific adaptation. For the FEG and PSG sets, we also performed functional enrichment analyses. The results will be favorable for understanding the divergent adaptation of the TP *Gynaephora* species to different high-altitude environments and provide new insights into high-altitude adaptation of animals.

## Result

### Illumina sequencing, assembly and annotation

**Figure 1:**
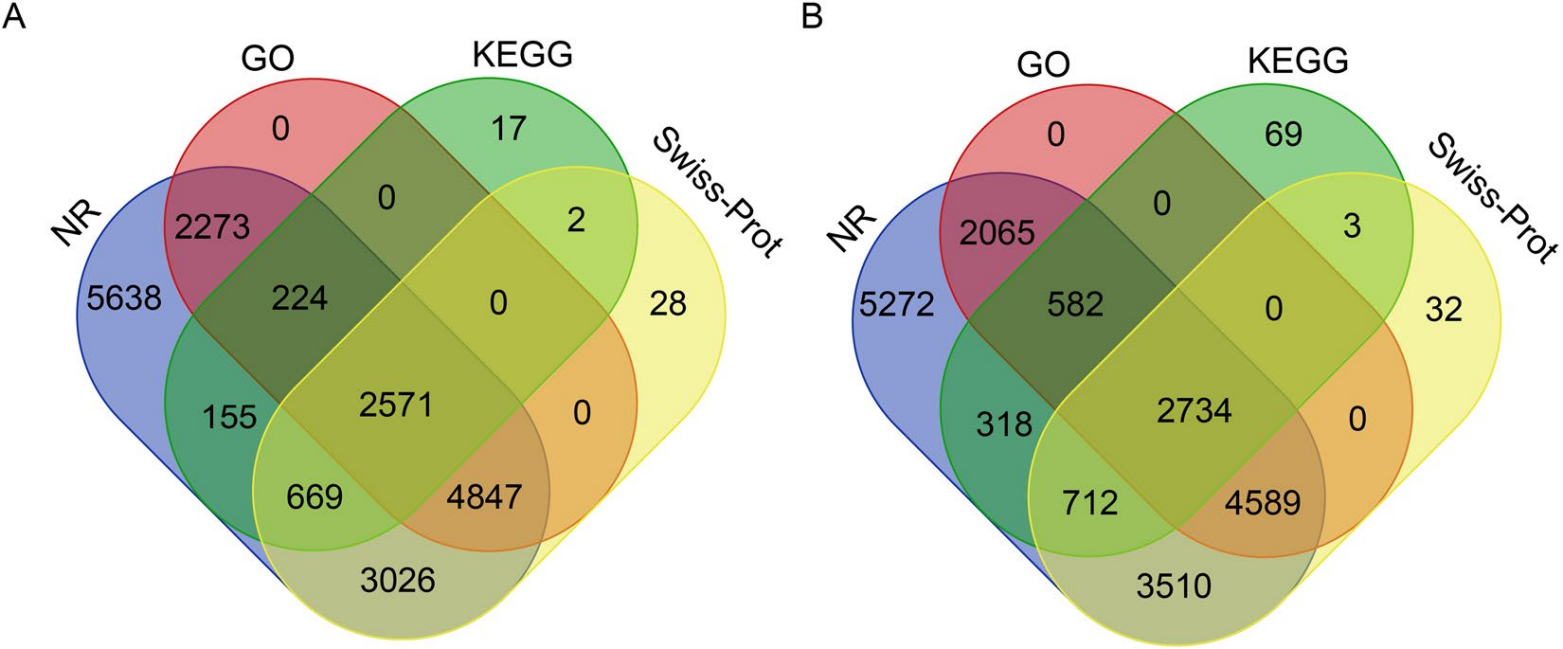
Venn diagrams of annotated information for *Gynaephora alpherakii* (**A**) and *G. menyuanensis* (**B**) unigenes among four databases.

Reads generated by sequencing were sufficient, indicated by an adequate sequencing saturation (Fig. S1). A total of ~4,810 million and ~4,990 million raw base pairs were generated for *G. alpherakii* and *G. menyuanensis*, respectively (Table 1). After removing low-quality reads, we obtained 3,700 and 3,830 million clean reads for the two *Gynaephora* species, respectively. After *de novo* assemblies, we obtained 35,593 sequences of unigenes and 28.53 mega base pairs (Mb) for the total length of the final assemblies for the *G. alpherakii* library. The N50 length of the library was 1,355 bp; the mean length of the assembly was 801 bp. For the *G. menyuanensis* library, we obtained 36,313 unigenes with total lengths of 29.05 Mb and the N50 of 1,334 bp; the mean length of the assembly was 799 bp. We aligned the reads of each sample back to the respective unigene set and found that 82.27% (*G. alpherakii*) and 83.16% (*G. menyuanensis*) of the reads were properly aligned, suggesting that the quality of RNA-seq and sample assemblies was reasonably good. The information of data production is shown in Table 1, and the length distribution of all unigenes is exhibited in Fig. S2. Analysis results of the software tool BUSCO (Benchmarking Universal Single-Copy Orthologs) based on 2675 near-universal single-copy orthologs in the arthropod gene set revealed that 66.3% of *G. alpherakii* genes were “complete”, 14.8% were “fragmented”, and the remaining genes were “missing” in *G. alpherakii*. For *G. menyuanensis*, 64.7% genes were “complete” and 13.9% were “fragmented”. For *L. dispar*, 61.4% genes were “complete” and 19.7% were “fragmented”. In comparison to 46 other arthropod species assemblies^27^, the BUSCO analysis showed that the quality of our assembled unigenes was better than that of the majority of transcriptome assemblies. A total of 19,450 unigenes (54.65%) of *G. alpherakii* and 19,886 unigenes (54.76%) of *G. menyuanensis* were successfully annotated to four databases: non-redundant (NR) protein database of GenBank, Swiss-Prot, kyoto encyclopedia of genes and genomes (KEGG) and gene ontology (GO). A larger number of unigenes were not only mapped to the NR but also to the other three databases, indicating that sequences of the two *Gynaephora* species were adequately annotated (Fig. 1).

**Table 1:** Summary of sequenced transcriptome data.

	*G. alpherakii*	*G. menyuanensis*
Raw data (Gb)	4.81	4.99
Use data (Gb)	3.7	3.83
Total number of reads	18,298,558	18,944,950
Read length (bp)	101	101
Total length of unigenes (Mb)	28.53	29.05
Total sequences of unigenes	35,593	36,313
N50 length of assembly (bp)	1,355	1,334
Mean length of assembly (bp)	801	799
Unigenes annotated	19,450	19,886

### Identification of putative orthologues

**Figure 2:**
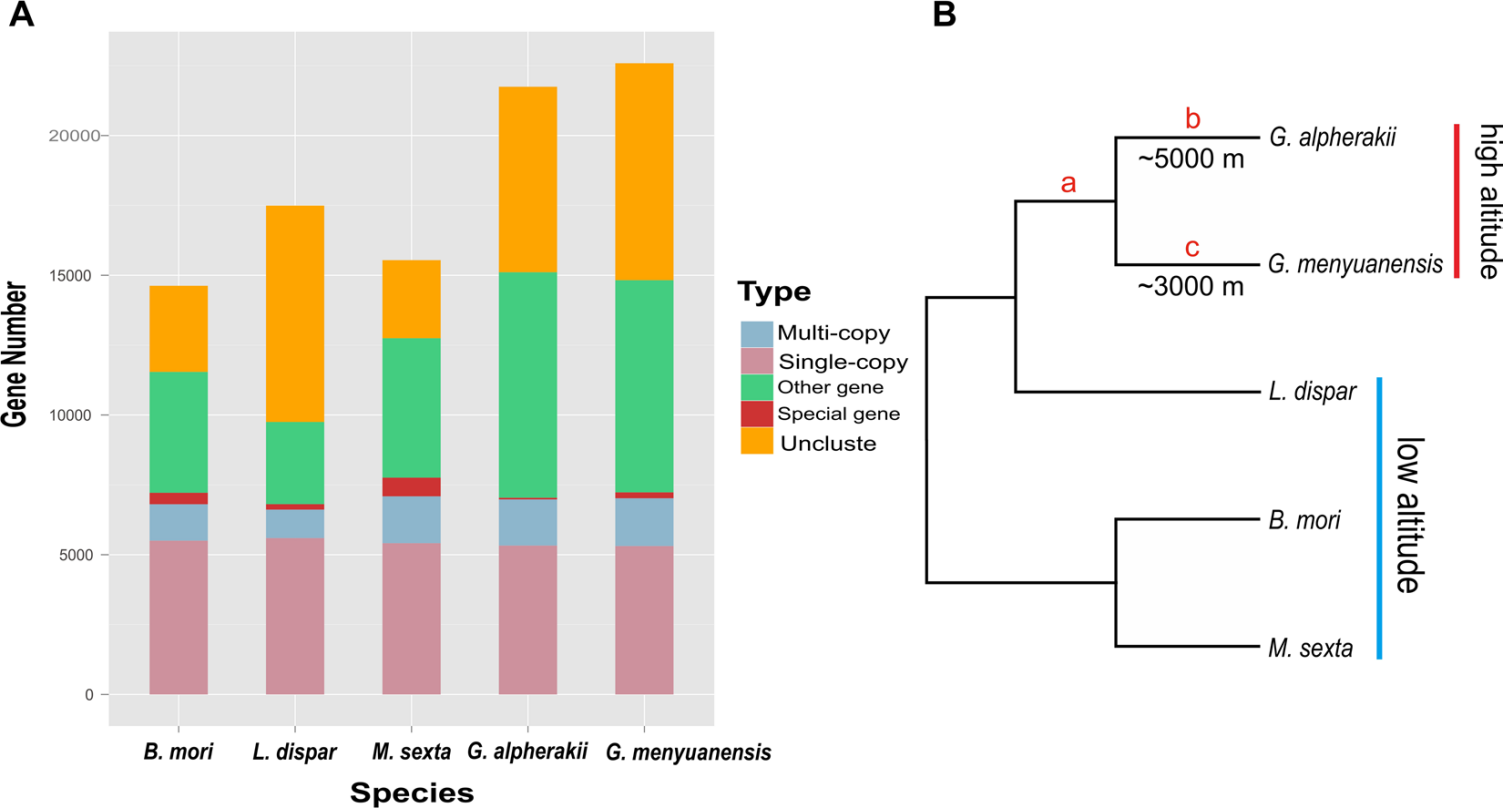
(**A**) Gene family clustering statistics. Muti-copy orthologs include the common orthologs with different copy numbers in the five species, single-copy orthologs include the common orthologs with the same number of copies in the five species, other orthologs include the unclassified orthologs, special orthologs include the genes belonging to gene families existing in only one species, and unclustered genes include the genes that cannot be assigned into gene families. (**B**) Tree topology of the five moths. This tree topology originated from previous phylogenetic analyses among these five species (*Gynaephora alpherakii*, *G. menyuanensis*, *Lymantria dispar*, *Bombyx mori*, and *Manduca sexta*)^24,54^. Branch a indicates the common ancestor of *G. alpherakii* and *G. menyuanensis*, branch b indicates *G. alpherakii*, and branch c indicates *G. menyuanensis*.

A total of 6,036 putative one-to-one orthologues were identified among the *G. alpherakii*, *G. menyuanensis*, *L. dispar, B. mori*, and *M. sexta* (Fig. 2A). After aligning and removing low quality and short sequences, 5,869 one-to-one genes were retained for the downstream analyses.

### Functional analysis of fast-evolving genes

A total of 34 and 26 FEGs were identified in *G. alpherakii* (branch b, Fig. 2B) and *G. menyuanensis* (branch c), respectively, whereas 24 FEGs in the common ancestor of *G. alpherakii* and *G. menyuanensis* (branch a) (Supplementary Dataset 1). Functional enrichment analyses showed that the FEGs (24) in the branch a was significantly enriched in functional categories associated with metabolism and enzyme activity, including “lipid particle”, “microtubule associated complex”, and “transferase activity”. The FEGs (34) in the branch b were significantly enriched in functional categories related to energy metabolism and oxidative stress (Table 2), e.g. “response to oxidative stress”, “oxidation-reduction process”, and “electron carrier activity”; moreover, KEGG enrichment analysis detected one significant pathway, “ribosome biogenesis in eukaryotes”. In the branch c, 26 FEGs were significantly enriched in GO terms involving transcription, enzyme activity and energy metabolism, e.g. “negative regulation of transcription from RNA polymerase II promoter”, “oxidation-reduction process”, and “acid phosphatase activity”.

**Table 2:** List of enriched GO terms and KEGG pathways from fast-evolving genes (FEGs) in the two *Gynaephora* species and their ancestral branch.

Classification	GO ID	Term	FDR
**Branch b (** ***G***. ***alpherakii*** **)**			
Biological Process	GO:0006979	Response to oxidative stress	0.0057
Biological Process	GO:0044710	Single-organism metabolic process	0.0059
Biological Process	GO:0055114	Oxidation-reduction process	0.0423
Cellular Component	GO:0005811	Lipid particle	0.0092
Cellular Component	GO:0005730	Nucleolus	0.0334
Cellular Component	GO:0005739	Mitochondrion	0.0372
Molecular Function	GO:0009055	Electron carrier activity	0.0662
	**KEGG ID**		
	ko03008	Ribosome biogenesis in eukaryotes	0.0444
**Branch c (** ***G***. ***menyuanensis*** **)**			
Biological Process	GO:0007350	Blastoderm segmentation	0.0001
Biological Process	GO:0000122	Negative regulation of transcription from RNA polymerase II promoter	0.0070
Biological Process	GO:0055114	Oxidation-reduction process	0.0238
Molecular Function	GO:0003993	Acid phosphatase activity	0.0001
**Branch a (The common ancestor of** ***G***. ***alpherakii*** **and** ***G***. ***menyuanensis*** **)**			
Cellular Component	GO:0005811	Lipid particle	0.0159
Cellular Component	GO:0005875	Microtubule associated complex	0.0243
Molecular Function	GO:0016740	Transferase activity	0.0123

### Identification of positively selected genes associated with hypoxia, cold and UV radiation

To identify key genes that may have a close association with the adaptation of *Gynaephora* to major ecological stressors (oxygen content, temperature and UV radiation) resulting from divergent high-altitude environments, we performed a two-step approach to screen all the candidate genes from 71 PSGs, 90 PSGs, and 82 PSGs identified in branches b, c, and a, respectively (Supplementary Dataset 2), according to previous studies^4,5,28^. First, we identified the GO categories involved in adaptation to major ecological stressors on the TP from previous studies, and then the candidate genes encompassing the functions and their associated GO categories for adaptation to major ecological challenge of high altitudes were considered as targets. A total of eight, three, and five genes were identified in the branches b, c, and a, respectively, using this method (Table 3). In the branch a, one gene encoded proteins associated with mitochondrial electron transport, i.e. mitochondrial-processing peptidase subunit beta (MPPB); two genes encoded proteins associated with pigment metabolic and pigment cell differentiation process, including GATA-binding factor A (GATA-A) and protein sidekick (SDK); the other genes were associated with response to oxidative stress (chloride intracellular channel exc-4, EXC4) and TOR signaling (regulatory associated protein of mTOR, RAPTOR). In the branch b, one gene encoded proteins related to single-organism metabolic (cytochrome b5, CYTB5); two genes encoded proteins associated with oxidation-reduction process (gamma-glutamyl phosphate reductase, GPR; probable saccharopine dehydrogenase [NADP(+), L-glutamate-forming], SDH); two genes encoded proteins involved in obsolete ATP catabolic process (protein scarlet, ST; ABC transporter F family member 4, ABCF4); particularly, gene encoding 3-hydroxyacyl-CoA dehydrogenase type-2 protein (SCU) participated in many biological function, including acyl-CoA metabolic process, oxidation-reduction process, and fatty acid metabolic process; the remaining two genes encoding DNA repair protein, rad50 (RAD50) and DNA repair protein xrcc (XRCC) were annotated to DNA repair and response to hypoxia, respectively. In the branch c, three genes associated with hypoxia, cold and UV radiation were annotated to GO terms involving cellular response to DNA damage stimulus (double-strand break repair protein, MRE11), obsolete ATP catabolic process (protein scarlet, ST), and oxidation-reduction process (gamma-glutamyl phosphate reductase, GPR).

**Table 3:** List of positively selected genes involved in response to hypoxia, cold and UV radiation in the two *Gynaephora* species and their ancestral branch. Stars (*) indicate insects-specific candidate genes identified according to our present understanding of the phenotypic adaptation of insects to high altitudes.

Protein homolog	FDR	GO terms	GO ID
**Branch b (** ***G***. ***alpherakii*** **)**			
Cytochrome b5, CYTB5	0.0000	single-organism metabolic process	GO:0044710
3-hydroxyacyl-CoA dehydrogenase type-2, SCU	0.0291	acyl-CoA metabolic process, oxidation-reduction process, fatty acid metabolic process	GO:0006637, GO:0055114, GO:0006631
Gamma-glutamyl phosphate reductase, GPR	0.0000	oxidation-reduction process	GO:0055114
Probable saccharopine dehydrogenase [NADP( + ), L-glutamate-forming], SDH	0.0000	oxidation-reduction process	GO:0055114
Probable chitinase 3, CHT3*	0.0000	chitin metabolic process	GO:0006030
Protein scarlet, ST	0.0000	obsolete ATP catabolic process	GO:0006200
ABC transporter F family member 4, ABCF4	0.0000	obsolete ATP catabolic process	GO:0006200
DNA repair protein, RAD50	0.0000	DNA repair	GO:0006281
DNA repair protein xrcc, XRCC	0.0098	response to hypoxia	GO:0001666
**Branch c (** ***G***. ***menyuanensis*** **)**			
Double-strand break repair protein, MRE11	0.0000	cellular response to DNA damage stimulus	GO:0006974
Ecdysone-inducible protein, E75*	0.0000	regulation of ecdysteroid metabolic process	GO:0007553
Protein scarlet, ST	0.0000	obsolete ATP catabolic process	GO:0006200
Probable chitinase 2, CHT2*	0.0000	chitin catabolic process	GO:0006032
Gamma-glutamyl phosphate reductase, GPR	0.0000	oxidation-reduction process	GO:0055114
**Branch a (The common ancestor of** ***G***. ***alpherakii*** **and** ***G***. ***menyuanensis*** **)**			
Mitochondrial-processing peptidase subunit beta, MPPB	0.0210	mitochondrial electron transport, ubiquinol to cytochrome c	GO:0006122
GATA-binding factor A, GATA-A	0.0136	pigment metabolic process	GO:0042440
Regulatory associated protein of mTOR, RAPTOR	0.0045	TOR signaling	GO:0031929
Protein sidekick, SDK	0.0314	pigment cell differentiation	GO:0050931
Chloride intracellular channel exc-4, EXC4	0.0204	response to oxidative stress	GO:0006979
Probable chitinase 3, CHT3*	0.0004	chitin metabolic process	GO:0006030

Second, according to our present understanding of the phenotypic adaptation of insects to ecological stressor, we identified one, two, and one candidate function-based PSGs in the branches b, c, and a, respectively. Of them, three genes with insect-related GO annotations were chitinase members (chitin metabolic process), including genes encoding probable chitinase 2 and 3 (CHT2 and CHT3). The remaining one was related to regulation of ecdysteroid metabolic process (ecdysone-inducible protein, E75). These four genes showed insect-related GO annotations and were associated with chitin formation and ecdysteroid metabolism in insects.

### Functional analysis of positively selected genes

To further understand the function and pathway of a set of PSGs, we enriched them to GO categories and KEGG pathways for each of three target branches (Table 4). In the branch c, only one GO term (“integral component of plasma membrane”) belonging to cellular components, and one KEGG pathway (“mTOR signaling pathway”) were significantly enriched. In the branch b, three GO terms were significantly enriched: “obsolete ATP catabolic process” (biological process), “mitochondrion” (cellular components) and “electron carrier activity” (molecular function). In the branch c, four significantly enriched GO terms (three and one terms belonged to biological process and molecular function, respectively) were obtained, including “metabolic process” and “acid phosphatase activity”.

**Table 4:** List of enriched GO terms and KEGG pathways from positively selective genes (PSGs) in the two *Gynaephora* species and their ancestral branch.

Classification	GO ID	Term	FDR
**Branch b (** ***G***. ***alpherakii*** **)**			
Biological Process	GO:0006200	Obsolete ATP catabolic process	0.0469
Cellular Component	GO:0005739	Mitochondrion	0.0404
Molecular Function	GO:0009055	Electron carrier activity	0.0357
**Branch c (** ***G***. ***menyuanensis*** **)**			
Biological Process	GO:0050794	Regulation of cellular process	0.0385
Biological Process	GO:0000122	Single-organism cellular process	0.0160
Biological Process	GO:0008152	Metabolic process	0.0381
Molecular Function	GO:0003993	Acid phosphatase activity	0.0226
**Branch a (The common ancestor of** ***G***. ***alpheraki*** *i* **and** ***G***. ***menyuanensis*** **)**			
Cellular Component	GO:0005887	Integral component of plasma membrane	0.0393
	**KEGG ID**		
	ko04150	mTOR signaling pathway	0.0472

## Discussion

The transcriptome represents a sample of the spatiotemporally-expressed genome and can be used as an entry into the genome divergence analysis under the lack of a sequenced genome, and most of transcriptomes likely contain targets of natural selection^16,29^. So far, studies of the mechanisms of divergent altitude adaptation have been conducted in different animals using the RNA-seq technology^28,30^. Previous studies have provided adaptive evidence to higher altitudes among different species^30,31^, but additional research across a wide range of animal groups is necessary to provide a more comprehensive understanding of the complexity of divergent altitude adaptation.

In this study, we explored how *Gynaephora* commonly adapt to the TP environments in the long-term evolutionary process by functional enrichment analysis for FEG and PSG sets in the ancestral branch of the two *Gynaephora* species (branch a). The results of enriched GO categories of FEGs indicated that functional GO categories related to enzymatic activity and lipid metabolism had evolved faster in the branch c relative to other low-altitude insects. This evidence of fast evolution provided important insights into the common mechanisms of high-altitude adaptation of *Gynaephora*, as observed in other TP animals, such as TP loach^30^ and plateau zokor^31^. Additionally, it is well-known that hypoxia, cold, and strong UV radiation are major challenge for TP animals in high-altitude habitats. Therefore, the extreme environments necessitate high energy metabolism, a strong resistance to UV and high-efficiency DNA repair, and hypoxia adaptation in species endemic to the TP^4,30^. Our results generated a list of PSGs in the branch a, which were indeed significantly enriched in functional categories and pathways associated with stability of plasma membrane and hypoxia response. Interestingly, hypoxia inducible factors-1 alpha (HIF-1α) protein expression is regulated by the mTOR signaling pathways, and this pathway promotes hypoxia adaptation of great tits to high altitude^32^. In the present study, PSGs were significantly enriched into mTOR signaling pathway, suggesting that mTOR signaling pathway could play a key role in hypoxia adaptation of *Gynaephora* species and mechanisms of hypoxia adaptation in insects showed a certain of similarity with vertebrates. Furthermore, some PSGs involving major ecological stressors (e.g., GATA-A and SDK associated with response to UV radiation; MPPB, RAPTOR and EXC4 with hypoxia) were identified. MPPB, a mitochondrial protein subunit, has been demonstrated that it was necessary for activity of mitochondrion function^33^ and higher mitochondrial activities promoted by MPPB may be required to meet the energy demand^34^. Rapamycin complex 1 (mTORC1) is a complex consisting of mTOR, a serine/threonine kinase, connecting with the RAPTOR, a regulatory associated protein of mTOR^35^. mTORC1 is an important regulator of cell growth and proliferation in response to hypoxia. mTORC1 activity could be strongly inhibited under hypoxia^35^, thus adaptive evolution of gene encoding RAPTOR protein may be helpful for remit inhibitory mTORC1 in *Gynaephora*. EXC4 is a member of chloride intracellular channels family. The previous study have demonstrated that overexpression of this gene family in human pulmonary artery endothelial cells increases endothelial cell response to oxidative stress by translocating to the cell periphery from cell nucleus and cytoplasm^36^. Despite GATA-A and SDK function remain not to be demonstrated by experiments, the GO annotation showed their potential role involved in pigment generation, thus they may be necessary for resisting UV radiation by pigment cell in TP *Gynaephora* species. Overall, our study exhibited adaptive evidence to harsh highland environments in the two *Gynaephora* species before they diverged at the early uplift of the TP.

After diverging from the common ancestor, *G. alpherakii* and *G. menyuanensis* have adapted to divergent high-altitude environments^29^. The result of functional enrichment analysis for FEGs was highly overlapping with that of PSGs. These categories indicated that high-altitude adaptation of *Gynaephora* species mainly involves several features, including fast evolutionary rates and positive selection on genes involved in hypoxia, ATP and energy metabolism, enzyme activity, mitochondrion, cellular process, as reported in other TP animals^30,31^. Significantly fast evolution of genes in energy metabolism and response to oxidative stress also provided the evidence that the *Gynaephora* obtained most energy by aerobic oxidation rather than anaerobic glycolysis, as observed in the plateau zokor^31^. In PSGs of the *G. alpherakii* (branch b) and *G. menyuanensis* (branch c) lineages, GO terms involved in energy metabolism, such as electron carrier activity and acid phosphatase activity, suggesting that this category of genes may be associated with divergent-altitude adaptation. Moreover, we screened several targeted PSGs involved in major ecological challenge from divergent-altitude environments (e.g., CYTB5, SCU, ABCF4, XRCC, GPR and ST associated with response to hypoxia; SDH with low temperature; RAD50 and MRE11 with UV radiation) in *G. alpherakii* and *G. menyuanensis* lineages. Specially, methemoglobin cannot bind to oxygen and caused tissue hypoxia at high concentrations, and then methemoglobin induction caused lower CYTB5 activity in *Salvelinus fontinalis* ^37^. SCU is a key dehydrogenase in fatty acid metabolic process, and the up-regulation of dehydrogenase in endothelial cells may be associated with their relative hypoxia tolerance^38^. Two PSGs, encoding the ABCF4 and XRCC proteins, may be also associated with hypoxia in grassland caterpillars. According to previous studies in other animals, the function of ABCF protein family was to regulate survival by protecting cells/tissues from protoporphyrin accumulation under hypoxia^39^. The XRCC gene from the GO terms “response to hypoxia” was well-studied in high-altitude adaptation of ranid frogs (*Rana* species)^4^. To date, there is no any report about the function of GPR and ST proteins in hypoxia response, but oxidation-reduction and obsolete ATP catabolic process annotated by these two proteins are key biological process to hypoxia response of animals^4,30^. Certainly, potential role speculated by this analysis for GPR and ST proteins in hypoxia tolerance need to be further investigated. Rubio *et al*. found that the enzyme activity and expression level of the gene encoding SDH could be increased when diapause eggs of *Bombyx mori* were exposed to low temperature^40^, implying that SDH may be useful for survival of *G. alpherakii* under lower temperatures. In addition, RAD50 and MRE11 were associated with response to DNA repair and DNA damage, and up-regulation of an operon encoding RAD50 and MRE11 homologs attributed to induction of recombinational repair under UV treatment^41^, as well as their high expression promoted rapid repair of DNA damage by restraining homologous recombination in *Haloferax volcanii* to avoid the low resolution caused by DNA end with multiple partners in DNA repair intermediates^42^. Thus, RAD50 and MRE11 may be responsible for adaptation of *Gynaephora* species to high UV radiation.

Previous studies have demonstrated that the genetic basis of adaptation is specific among different animal groups, such as Tibet fish^30^, reptiles^16^, amphibian^4^ and birds^32^. Therefore, we particularly discussed specific adaptive mechanisms of the two *Gynaephora* species to different altitude environments. In the present study, several candidate genes with characteristics of insect groups were detected and their functions were related to ecdysteroid metabolism, chitin catalysis and metabolism. These genes may be involved in the unique response of insects to hypoxia and cold. E75 protein is a component of the organic matrix of calcium storage structures during the exoskeleton formation in arthropods^43^. A empirical finding supports that most insects develop smaller body sizes in hypoxia, which may be partly explained by biomechanical constraints imposed by the exoskeleton during moulting^44^. Given we have observed smaller body size of *G. alpherakii* than *G. menyuanensis* in our field investigation, suggesting that E75 may be useful for hypoxia adaptation of *Gynaephora* by decreasing their body size. CHT2 and CHT3 are two members of the chitinase family. Cai *et al*. reported low-temperature inducible expression and cold resistance of genes encoding chitinase in the desert beetle *Microdera punctipennis* ^45^. These insect-specific PSGs provided some valuable information for understanding uniquely adaptive mechanisms in insects endemic to the TP. Notably, ST and GPR proteins were shared in the list of PSGs between the two *Gynaephora* species (branches b and c), indicating that they may have experienced positive selection persistently under variable hypoxia with a rising altitude. Besides, PSGs encoding chitinase family could be detected among three branches, indicating key roles of chitinase in high- and divergent- altitude adaptation of TP *Gynaephora* species. The other PSGs involving adaptation to hypoxia, cold and UV radiation had also experienced alternatively adaptive evolution in *G. alpherakii* and *G. menyuanensis*, which could predominantly promote divergent adaptation of *Gynaephora* to different altitudes.

In summary, we for the first time explored the genetic basis of adaptation of *Gynaephora* to divergent altitudes on the TP at the genome scale. The identified FEGs and PSGs, combined with their functional terms enriched, provided evidence for high-altitude adaptations of the common ancestor of *G. alpherakii* and *G. menyuanensis*, and divergent-altitude adaptation between the two *Gynaephora* species. Particularly, functional terms enriched by FEGs were highly overlapping with those enriched by PSGs, indicating that our analysis was robust and reliable, and the identified candidate genes were extremely key for adaptive evolution of *Gynaephora* under altitude stress. Furthermore, some PSGs involved in response to hypoxia, cold and UV radiation were detected, which may contribute to high-altitude adaptation of the two *Gynaephora* to divergent ecological factors resulting from different high altitudes. This study provides genetic resources for the two TP *Gynaephora* species, which will open a window for further understands the high-altitude adaptation of insects at both molecular and phenotypic levels. Further study is needed to confirm the candidate genes identified in this study by population genetic and functional genomic approaches. Sequencing and comparative analyses for other *Gynaephora* species from the TP and other regions could further reveal adaptive basis of grassland caterpillars to different altitude environments on the TP.

## Methods

### Sampling, RNA extraction and Illumina sequencing

We collected *G. alpherakii* from Naqu County of Tibet (4,800 masl, 31°48′N/92°04′E, 57% of the oxygen available at sea level (partial oxygen pressures calculated based on formula used in^20^), annual average temperature ~−3 °C), and *G. menyuanensis* from Menyuan County, Qinghai Province (3,000 masl, 37°62′N/101°19′E, 78% of the oxygen available at sea level, annual average temperature ~3 °C), on the TP. Nine different developmental stages, including egg, 1–6 larva instar, pupa and female adult, were sampled for each species. All of the samples were initially preserved by RNA later stabilization solution (Thermo Scientific, USA) in the field and transferred to −80 °C until used for RNA extraction. In order to avoid sampling randomness and local environmental noise, we continuously collected samples from three sampling sites (collected sites at least 70 kilometers apart) at the same altitudes for each developmental stage. Larval instars were determined according to the method of Yan *et al*.^46^. For each sample, total RNA was extracted from 10 individuals for larvae and adults or from approximately 90 eggs, including individuals from different sampling sites, using Trizol reagent (Thermo Scientific, USA) following the manufacturer’s protocol. Residual genomic DNA was digested by RNase-free DNase (Qiagen, Germany). The RNA concentration was quantified using a NanoDrop ND1000 spectrophotometer (Thermo Scientific, USA). RNA structural integrity was verified by 1.5% agarose gel electrophoresis and analyzed by an Agilent 2100 bioanalyser (Agilent Technologies, USA). 10 RNA samples of each group/sample were pooled together for further experiments. Next, to cover development-specific transcripts in *Gynaephora* species, above-pooled RNA was pooled equally from the nine developmental stages for RNA sequencing of each species. RNAs with a poly (A) tail were purified from the total RNA by NEBNext Poly(A) mRNA Magnetic Isolation Module (NEB, USA) and then cut into short sequences. Two cDNA libraries were constructed via NEBNext mRNA Library Prep Master Mix Set for Illumina (NEB, USA) and NEBNext Multiplex Oligos for Illumina (NEB, USA) according to the manufacturer’s instructions. The constructed library was sequenced using the Illumina HiSeq. 2000 platform at Biomarker Technologies Company (Beijing, China). The entire process followed a standardized procedure and was monitored by Biomarker’s Quality Control System.

### *De novo* assembly and annotation

The raw sequence reads were trimmed using Trimmomatic (v0.32) with a default parameter (http://www.usadellab.org/cms/?page=trimmomatic) by removing adapter sequences, sequences with unknown bases (N) >5% and low-quality bases (quality value <20). We used Prinseq (v0.20.4, http://prinseq.sourceforge.net/) subsequently to discard poly-A/T tails and low-complexity reads. Additionally, Deconseq (v0.4.3, http://deconseq.sourceforge.net/) was used to remove potential contaminated reads from sources like rRNA, human, bacteria, and virus. The reference of sources sequences was downloaded from FTP server of NCBI (ftp://ftp.ncbi.nih.gov/).

The *de novo* transcriptome assembly of clean reads of each data set, including *G. alpherakii*, *G. menyuanensis* and *L. dispar* (SRX371346 in SRA database of NCBI), was performed using Trinity software^47^ (running parameter: Trinity.pl–seqType fq–JM 50 G–left reads_1.fq–right reads_2.fq–CPU 6). We then used CD-HIT-EST^4^ (parameter: -c 0.95 –n 8) and TGICL pipeline^48^ with a minimum overlap length of 100 bp to produce a final unigene set by integrating sequence overlaps and eliminating redundancies. To gain function annotation information for the unigenes, the blast tool with default parameters (E-value = 10^−5^) was used to hit unigenes to public databases, including NR, Swiss-Prot, KEGG and GO. To assess quality and completeness of the *de nove* assemblies, we compared the transcriptomes obtained in the present study with a core set of arthropods using BUSCO (Benchmarking Universal Single-Copy Orthologs) v3 software with default settings (http://busco.ezlab.org/).

### Construction of one-to-one orthologous genes

Trinity can distinguish potential isoforms (formed by alternative splicing) from the same unigene, and these isoforms were marked by the same prefix. In this case, the longest isoform for each unigene was selected as a unique representation. The open reading frames (ORFs) of each unigene were predicted using TransDecoder as part of the Trinity package^47^. The longest ORF of each reference gene was extracted to maintain more sequence information and to represent unique gene. We reserved only the predicted coding sequences (CDSs) longer than 300 bp under a strict criterion and then obtained translated protein sequences for downstream analyses. Subsequently, we employed OrthoMCL^49^ with default parameter to identify orthologous groups (families) among five species: *G. alpherakii*, *G. menyuanensis*, *L. dispar*, *B. mori*, and *M. sexta*. This program takes all-against-all BLASTp as input and defines putative pairs of orthologs based on the best reciprocal BLAST hit (BRH) method. If the orthologous groups only contained one sequence from each of all species, this gene cluster was extracted as putative one-to-one orthologous. Moreover, the CDSs of the predicted orthologous genes were aligned by PRANK with “-F -codon” parameter^50^. Aligned sequences with ambiguous alignments were removed in further analysis. A manual check was also conducted to correct potential errors.

### Identification and function analysis of fast evolving genes and positively selected genes

To identify FEGs in the foreground branches, we used codeml program with the branch model in the PAML package^51^ (ver. 4.3). The null model assumed that all branches evolved at the same rate (the same *ω* value), and the alternative model allowed for the foreground branch (branches a, b, and c; Fig. 2B) to evolve under a different rate. A likelihood ratio test (LRT, df = 1) was applied to determine significant differences between alternative models for each orthologs. The false discovery rate (FDR) in multiple pairwise comparisons was used to correct significance levels of a likelihood ratio output from PAML in R (http://www.R-project.org). The genes with an FDR-adjusted *P*-value < 0.05 and a higher omega value in the foreground branch than in the background branches were considered as FEGs.

To detect positively selective signals of the one-to-one orthologous genes in branches a, b, and c of our guided tree (Fig. 2B), we implemented codeml program using the branch-site model with the optimized parameters according to the PAML manual (http://abacus.gene.ucl.ac.uk/software/pamlDOC.pdf). The branch-site model can detect positive selection that affects only a few sites on a target (foreground) branch of the species tree. LRT tests were performed by comparing two models: one allows sites to be under positive selection on the foreground branch, whereas another assumes that sites are evolving neutrally and under purifying selection. Their *P-*values were used to test significant level of the LRT values, and less than 0.05 was considered as target. Furthermore, the false discovery rate (FDR) in multiple pairwise comparisons was used to correct significance levels of *P*-values of all PSGs, with FDR-adjusted *P*-value < 0.05. The Bayes empirical Bayes (BEB) method was used to calculate posterior probabilities and to record positively selected sites (those with BEB value >0.95 were considered as target). The PSGs in each of target branches were subjected to further functional enrichment analyses.

The GO enrichment for target gene sets was performed and Fisher’s exact test was used to estimate significance of each term in Blast2GO pipeline^52^ (E-value = 10^−5^). Significance levels of all GO terms were corrected by controlling the FDR in multiple pairwise comparisons. KEGG enrichment analysis was performed using KOBAS 2.0^53^. We consider terms and pathways with significant value (FDR < 0.05) to be targets.

### Data availability

Raw Illumina reads of two *Gynaephora* species have been submitted to NCBI SRA (accession numbers SRR4242137 and SRR4242138).

## Electronic supplementary material

Supplementary Information

Supplementary Dataset 1

Supplementary Dataset 2
