# Supplementary material for: Comparative transcriptomic analysis of Tibetan Gynaephora to explore the genetic basis of insect adaptation to divergent altitude environments
Source: Sci Rep. 2017 Dec 5;7:16972. doi: 10.1038/s41598-017-17051-4 (PMC5717227; doi:10.1038/s41598-017-17051-4)
Supplement: Supplementary file 1 — Supplementary Information [file 41598_2017_17051_MOESM1_ESM.pdf]

Supplementary Information

Title: Comparative transcriptomic analysis of Tibetan *Gynaephora* to explore the genetic basis of insect adaptation to divergent altitude environments

Author list: Qi-Lin Zhang[1,2, †], Li Zhang[1, †], Xing-Zhuo Yang[1], Xiao-Tong Wang[1], Xiao-Peng Li[1], Juan Wang[1], Jun-Yuan Chen[2,*] & Ming-Long Yuan[1,*]

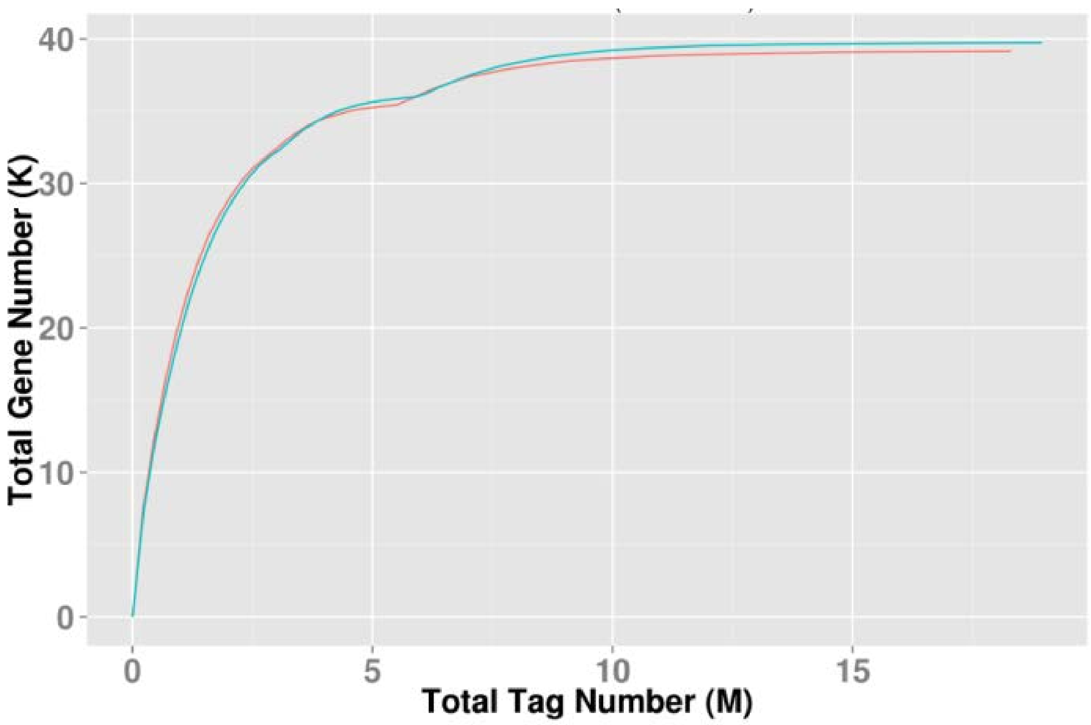

Figure S1. Map of sequencing saturation analysis. Red lines indicate *G. alpherakii*; Blue lines indicate *G. menyuanensis.* X-axis indicates number of reads obtained by sequencing; Y-axis indicates number of expressed genes. Genes with RPKM values > 0.1 were considered as expression.

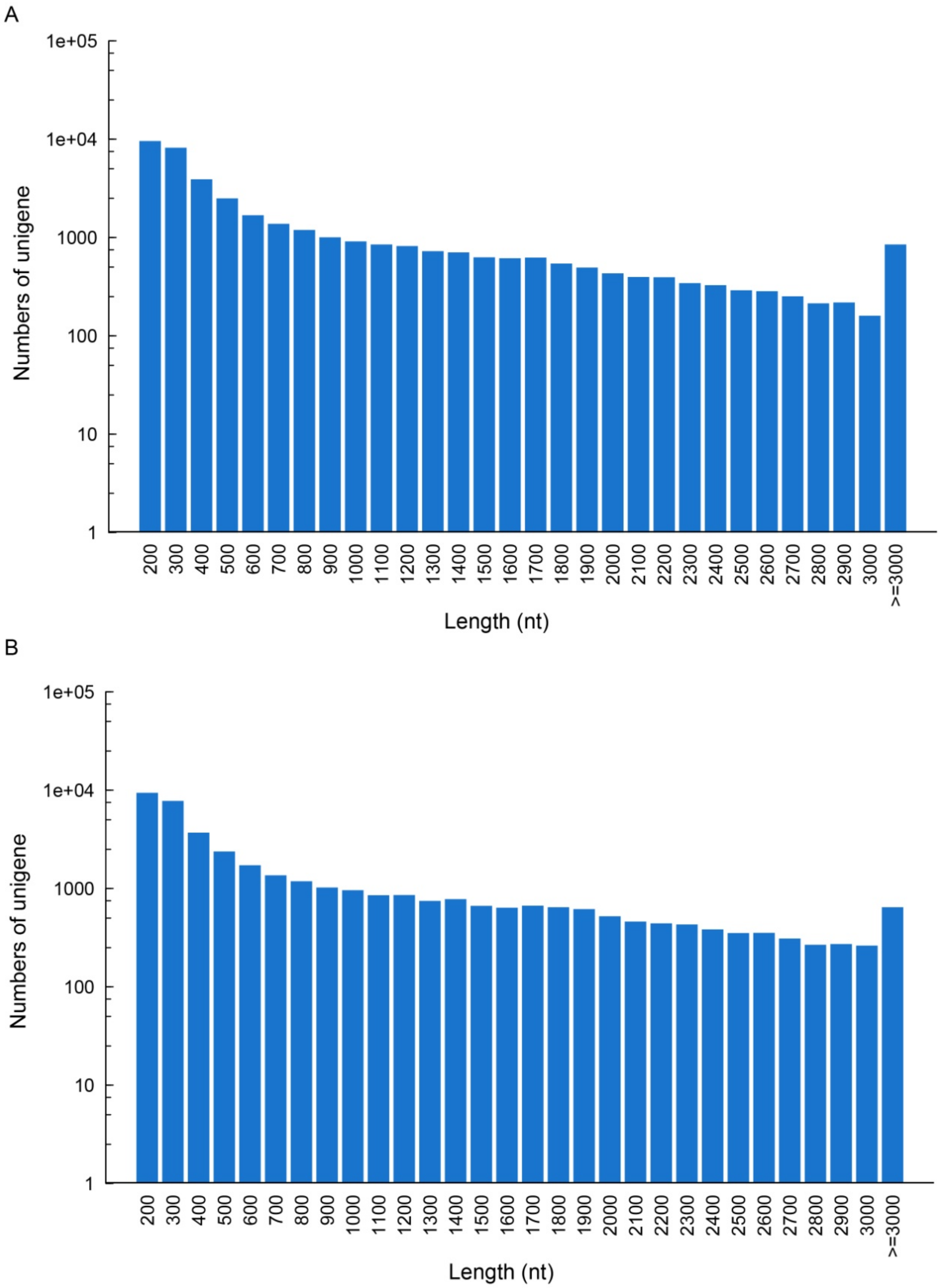

Figure S2. The length distribution of *G. alpherakii* (A) *G. menyuanensis* (B) unigenes. The horizontal coordinates are unigene lengths and the vertical coordinates are numbers of unigenes.
